# Supplementary material for: Light-driven self-assembly of spiropyran-functionalized covalent organic framework
Source: Nat Commun. 2023 Jun 23;14:3765. doi: 10.1038/s41467-023-39402-8 (PMC10290075; doi:10.1038/s41467-023-39402-8)
Supplement: Supplementary file 1 — Supplementary Information [file 41467_2023_39402_MOESM1_ESM.pdf]

Supplementary Information

Light-Driven Self-assembly of Spiropyran-Functionalized Covalent Organic Framework

Gobinda Das,[1] Thirumurugan Prakasam,[1] Nour Alkhatib,[1] Rasha G. AbdulHalim,[1] Falguni Chandra,[2] Sudhir Kumar Sharma,[3] Bikash Garai,[1,4] Sabu Varghese,[5] Matthew A. Addicoat,[6] Florent Ravaux,[7] Renu Pasricha,[5] Ramesh Jagannathan,[3] Na'il Saleh,[2,8] Serdal Kirmizialtin,[1,9] Mark A. Olson,*[10] and Ali Trabolsi*[1,4]

[1] Chemistry Program, New York University Abu Dhabi (NYUAD), Saadiyat Island, United Arab Emirates

[2] Chemistry Department, College of Science, United Arab Emirates University, P.O. Box 15551, Al-Ain, United Arab Emirates

[3] Engineering Division, New York University Abu Dhabi (NYUAD), United Arab Emirates

[4] NYUAD Water Research Center, New York University Abu Dhabi (NYUAD), Saadiyat Island, United Arab Emirates

[5] CTP, New York University Abu Dhabi, 129188 Abu Dhabi, UAE

[6] School of Science and Technology, Nottingham Trent University, Clifton Lane, NG11 8NS Nottingham, U.K.

[7] Quantum research center, Technology Innovation Institute, P.O. Box 9639, Abu Dhabi, United Arab Emirates

[8] Zayed Center for Health Sciences, United Arab Emirates University, P.O. Box 15551, Al Ain, United Arab Emirates

[9] Center for Smart Engineering Materials, New York University Abu Dhabi (NYUAD), United Arab Emirates

[10] Department of Physical and Environmental Sciences, Texas A&M University Corpus Christi, 6300 Ocean Dr., Corpus Christi, TX 78412 USA

These authors contributed equally: Gobinda Das, Thirumurugan Prakasam

Corresponding Authors

* ali.trabolsi@nyu.edu, mark.olson@tamucc.edu

Key words: Covalent organic framework, spiropyran, photoresponsive, self-assembly, vesicles.

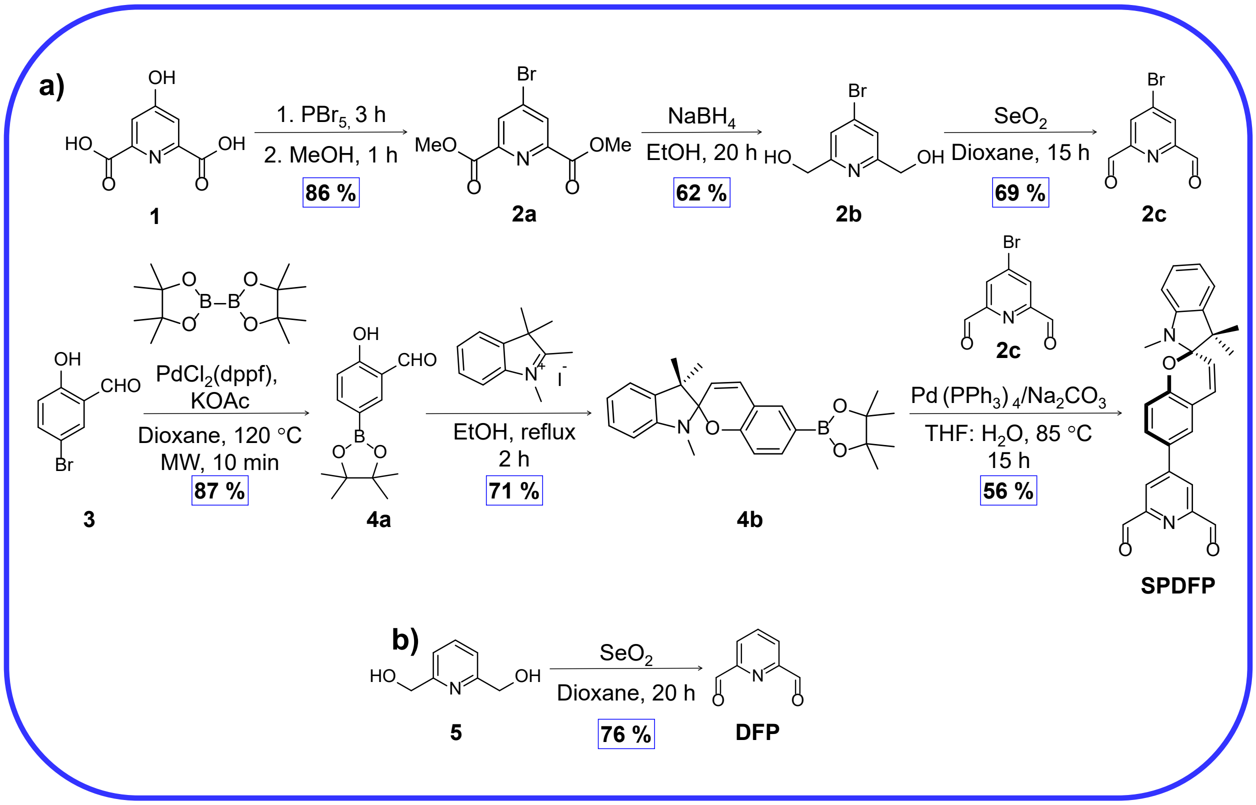

Supplementary Fig. 1. Synthetic route for the preparation of a) 4-(1',3',3'-trimethylspiro[chromene-2,2'-indolin]-6-yl)pyridine-2,6-dicarbaldehyde (SPDFP) and b) pyridine-2,6-dicarbaldehyde (DFP)

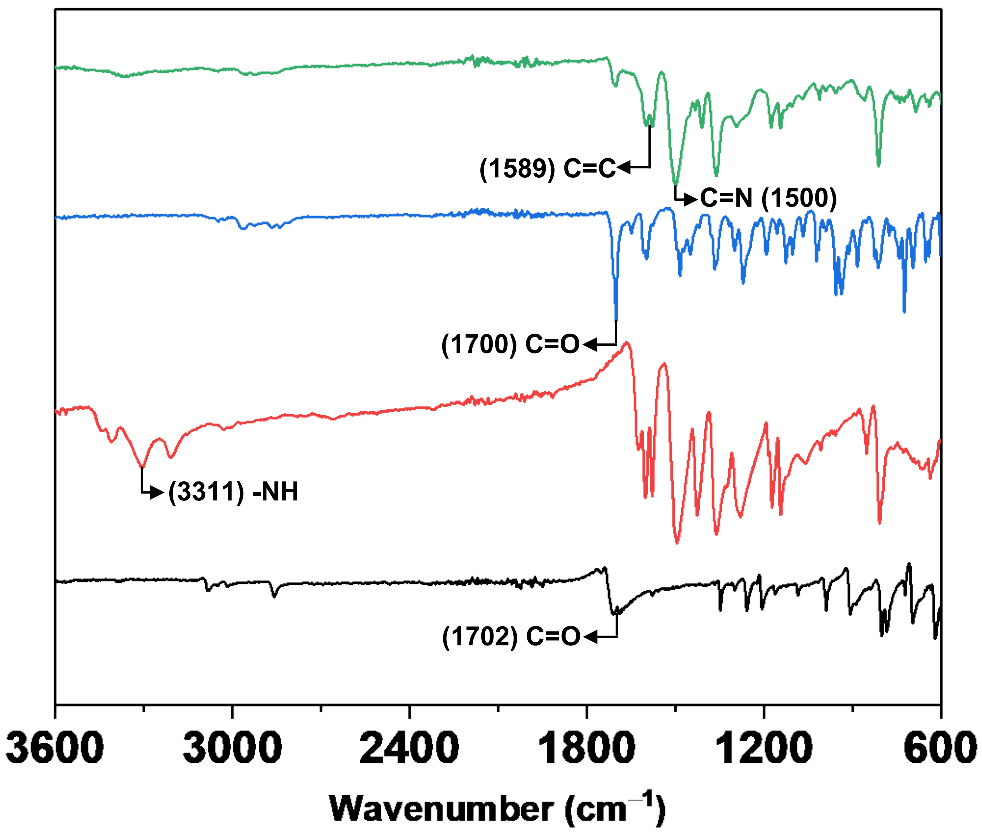

Supplementary Fig. 2. Stacked FT-IR spectra of TTA-SPDFP COF (green) and its precursors SPDFP (blue), TTA (red), and DFP (black).

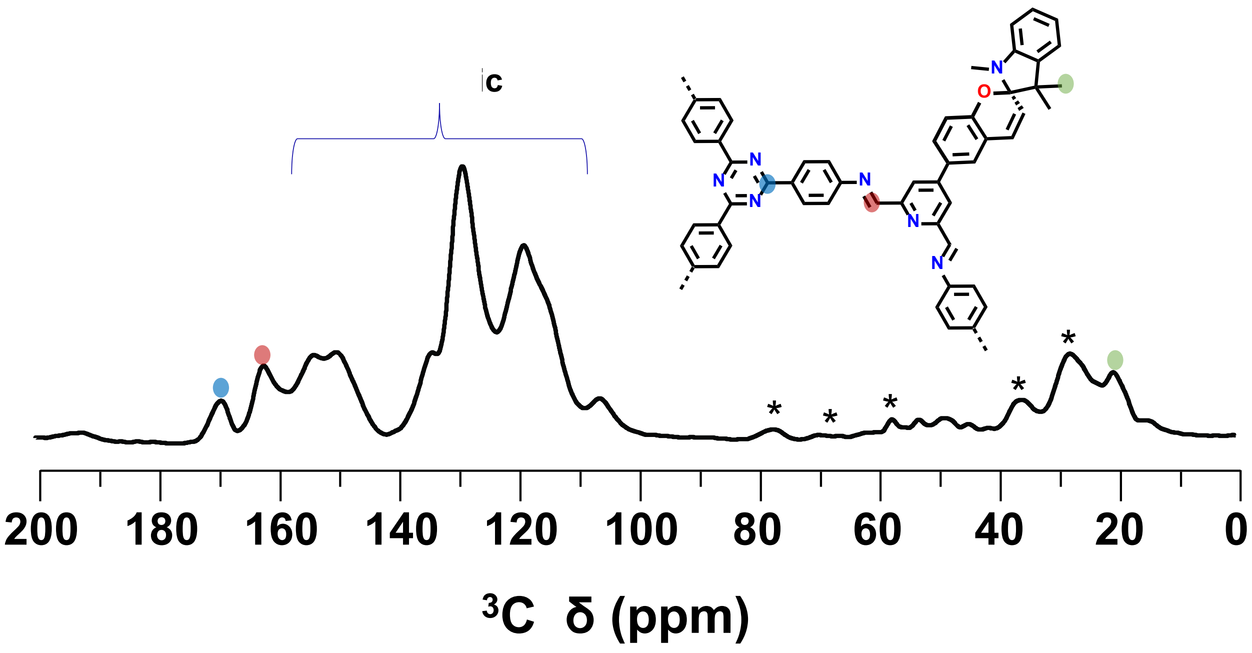

Supplementary Fig. 3. One-dimensional ^{13}C CPMAS spectrum of TTA-SPDFP COF. Spinning side bands are labelled with asterisks.

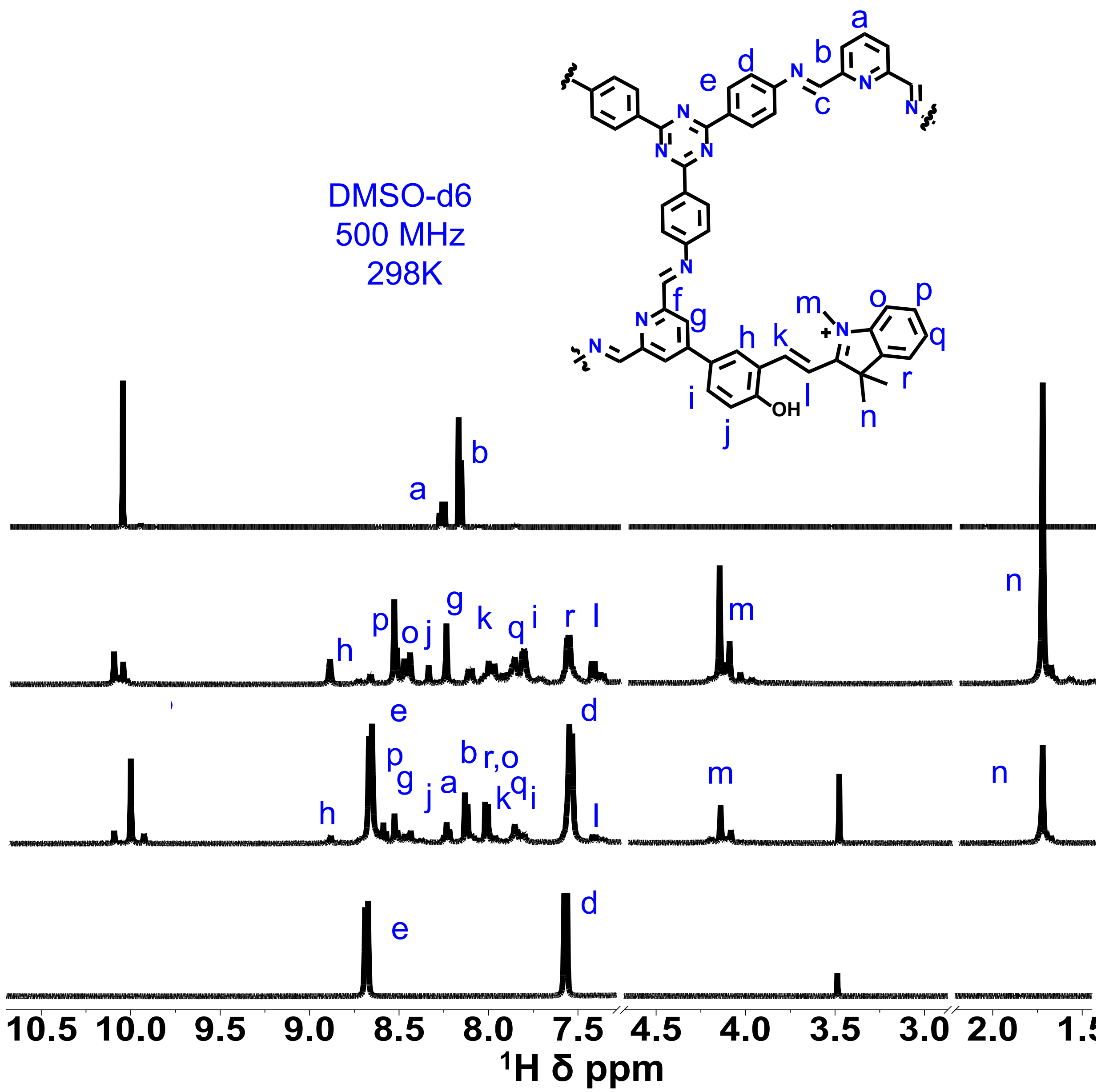

Supplementary Fig. 4a. ^{1}H-NMR spectrum of the digested TTA-SPDFP compared to ^{1}H-NMR spectra of the starting materials. This study was used to determine the monomer-linker ratio. To determine the linker ratio, the TTA-SPDFP COF was digested in 65% HNO_3 (aq) solution.

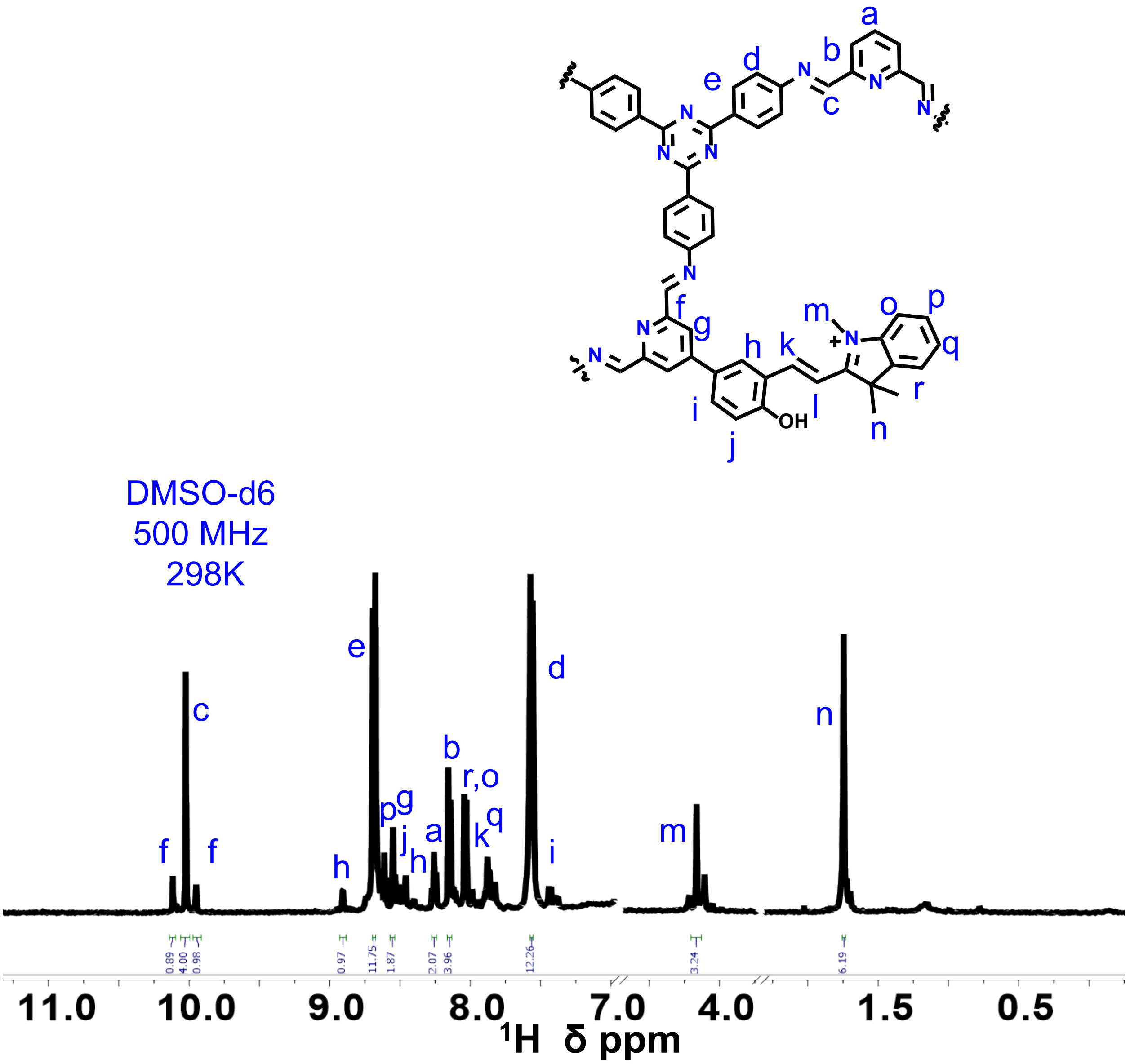

Supplementary Fig. 4b. Integration of the ^{1}H-NMR spectrum of the digested TTA-SPDFP COF used to determine the monomer-linker ratio.

Supplementary Table 1: Analysis of proton integration of the acid digested TTA-SPDFP COF

TTA-SPDFP COF linker Proton	Proton integration of the TTA-SPDFP after acid digestion			No. of protons per molecule	Linker ratio
	DFP	SPDFP	TTA		
Aromatic protons (b and g) at δ 8.41, and 8.19 ppm	4	1.9		2	DFP: SPDFP 2:1
Aromatic protons (e and d) at 8.61, and 7.54 ppm			24.1 (11.8+12.3)	12	TTA 2
Aldehyde protons (c and f) at δ 10.01, 10.10 and 9.95 ppm	4	1.9 (0.9+1)		2	DFP: SPDFP 2:1
Aliphatic protons (m and n) δ 4.16, 10.10 and 1.74 ppm		3.2 and 6.2		3 and 6	SPDFP 1
			Overall linker ratio		TTA: DFP: SPDFP 2:2:1

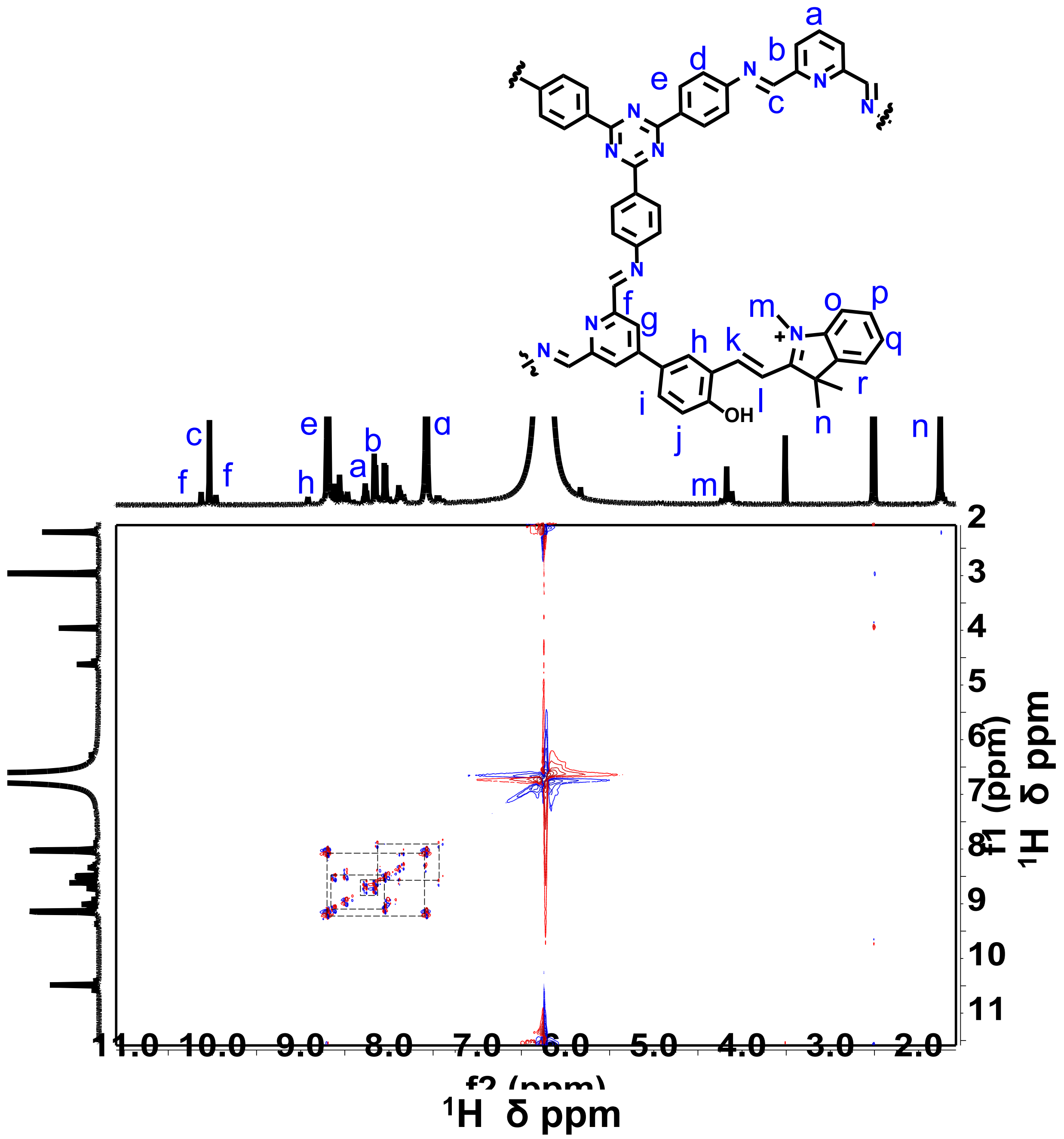

Supplementary Fig. 5. 2D ^{1}H-^{1}H COSY NMR spectrum of TTA-SPDFP COF after acid digestion.

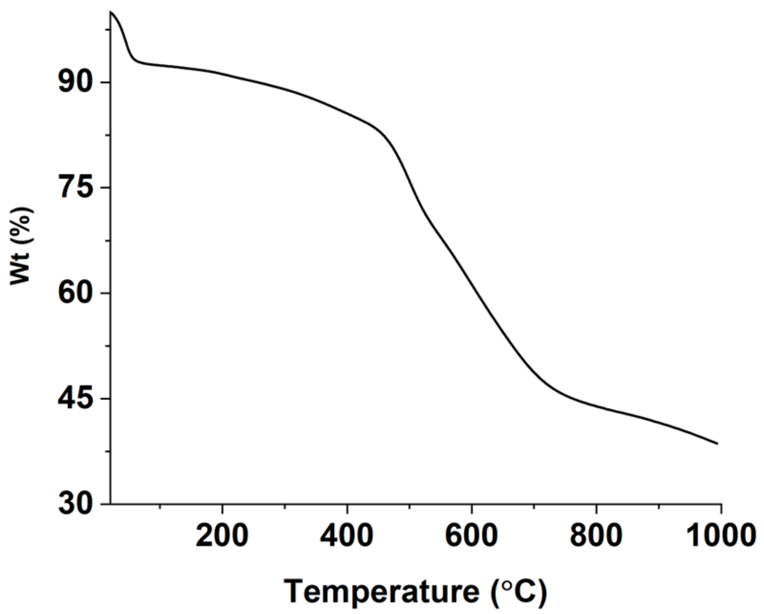

Supplementary Fig. 6. Thermogravimetric analysis of the as-synthesized TTA-SPDFP COF.

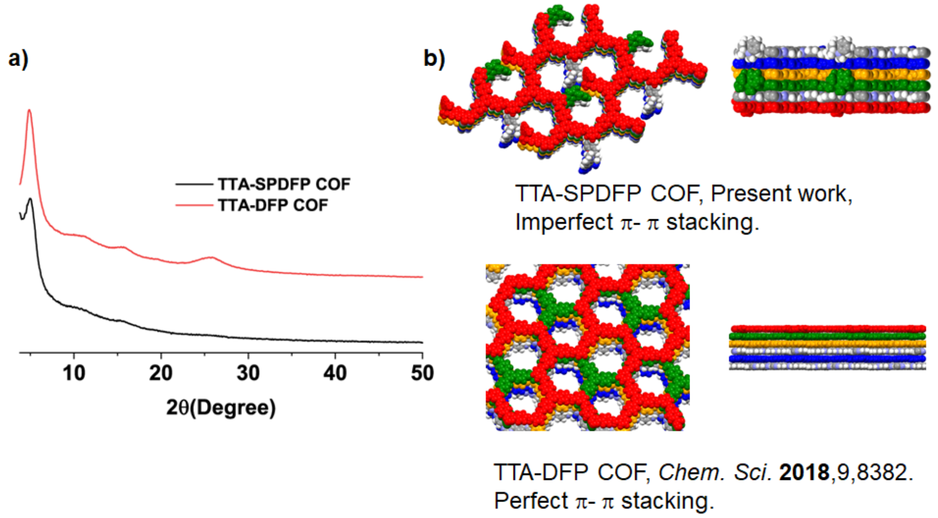

Supplementary Fig. 7. a) Comparison of the PXRD patterns of TTA-SPDFP and TTA-DFP. b) Simulated structures that resulted from an eclipsed AA stacking model for TTA-SPDFP and TTA-DFP. Weak PXRD intensity for TTA-SPDFP could be due to the distortion of the π−π stacking between the COF layers.

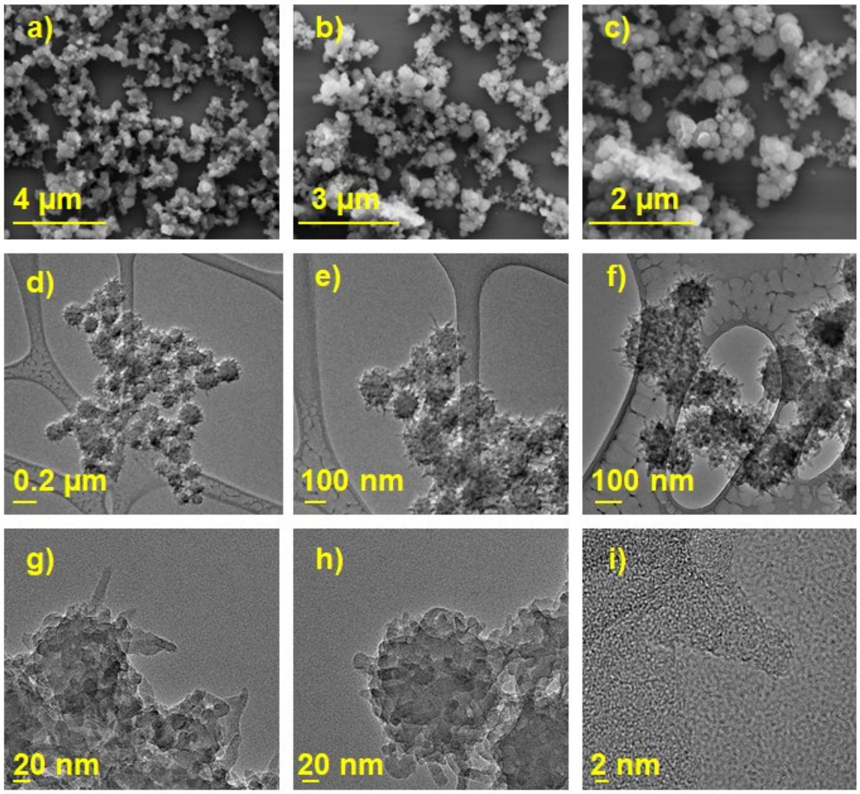

Supplementary Fig. 8. a-c) SEM and d-i) HRTEM images of TTA-SPDFP COF at various magnifications. For SEM analysis the sample was prepared by drop casting the diluted aqueous solution of the COF material on the Si wafer. For HRTEM analysis, the sample was prepared by drop casting the diluted aqueous solution on a holey carbon film mounted on a copper grid.

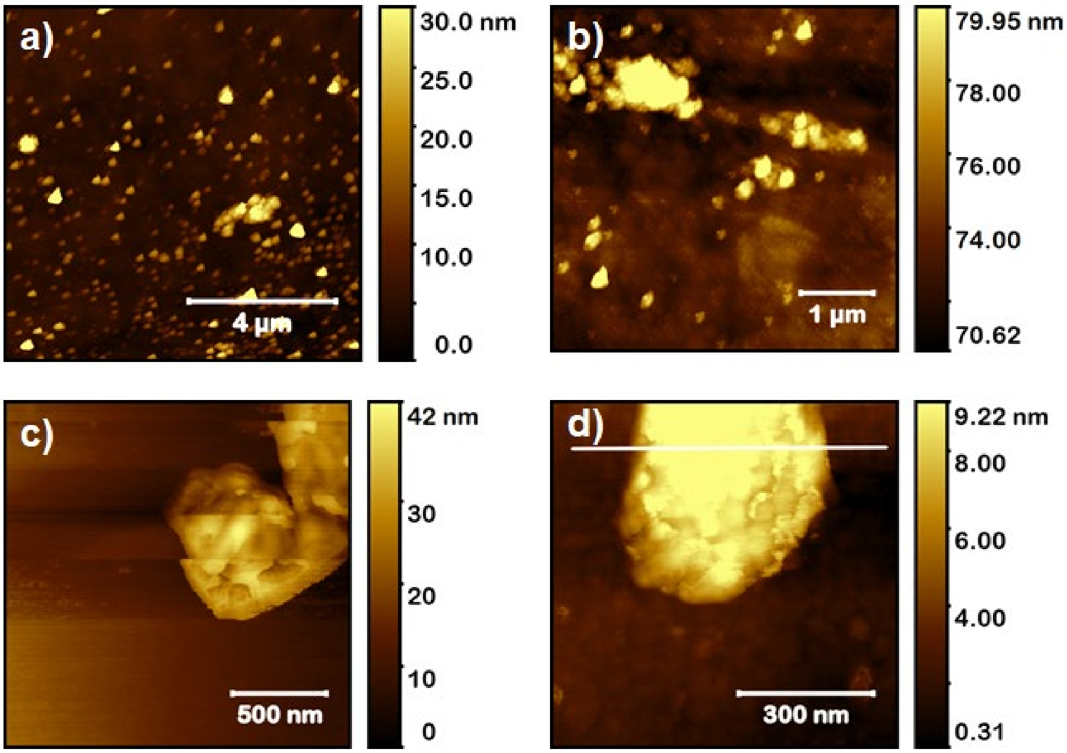

Supplementary Fig. 9. a-d) AFM images of TTA-SPDFP COF at different magnifications. The images were recorded by drop casting the diluted aqueous dispersed solution of TTA-SPDFP COF on Si wafer surface.

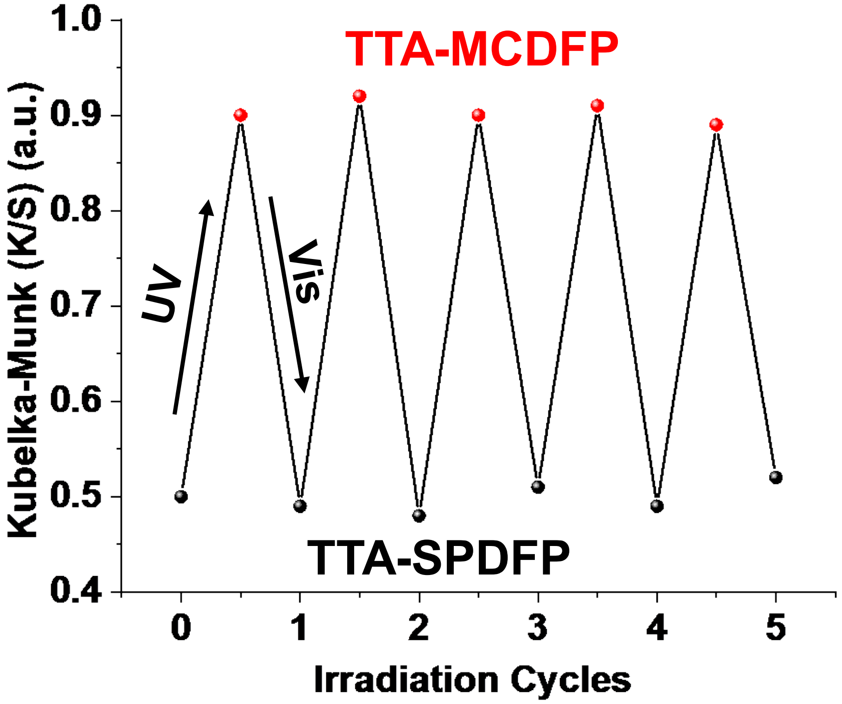

Supplementary Fig. 10. Reversible switching cycles of TTA-SPDFP COF in response to UV (365 nm) and visible (450 nm) lights. All cycles were performed at room temperature on a solid sample.

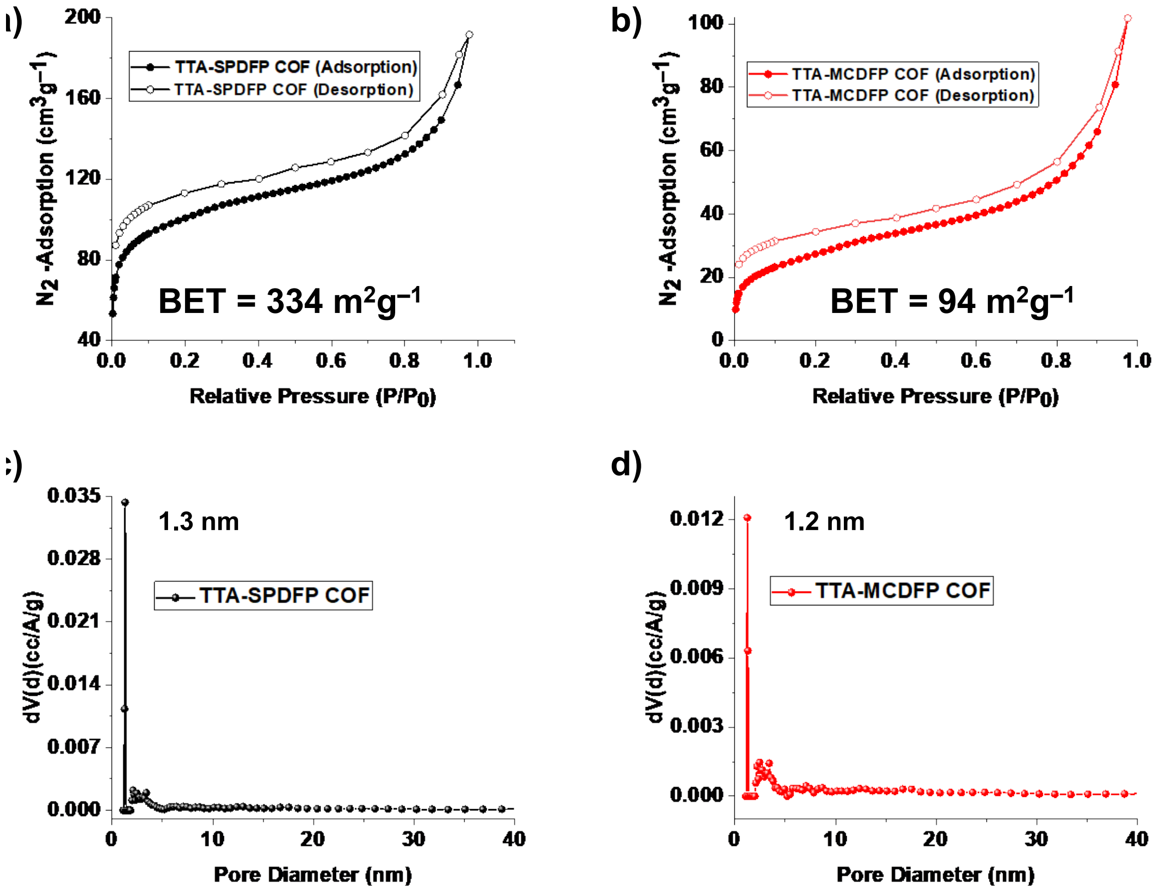

Supplementary Fig. 11. a-b) N_2 adsorption isotherms, and c-d) pore size distribution of TTA-SPDFP and TTA-MCDFP COFs. Surface area measurements were performed by activating the samples at 85 °C for 24 hours under high vacuum.

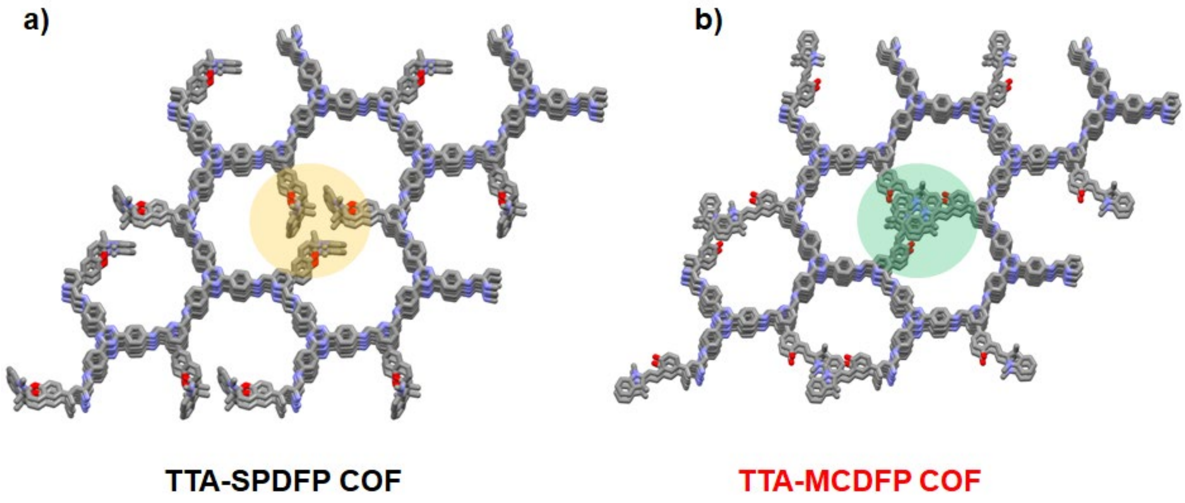

Supplementary Fig. 12. Structural differences between a) TTA-SPDFP, and b) TTA-MCDFP COFs. The yellow and green circles highlighting the light-induced changes inside the pore.

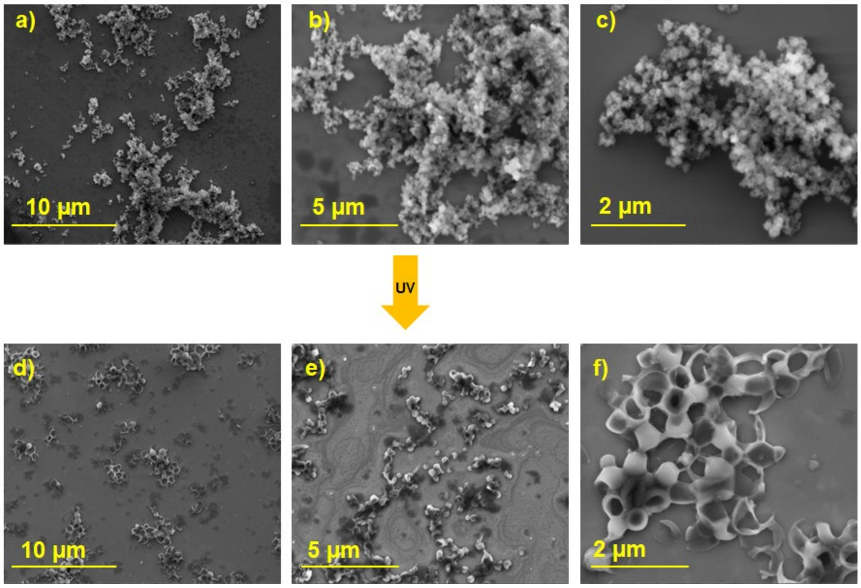

Supplementary Fig. 13. SEM images of TTA-SPDFP COF before a-c) and after d-f) photoirradiation. The two samples (TTA-SPDFP/MCDFP) were prepared by drop casting a dilute aqueous solution onto the Si wafer. TTA-SPDFP was first dispersed in water at room temperature and photochemical conversion was achieved after one hour photoirradiation (365 nm lamp). The photochemical conversion and microscopic analysis were performed at room temperature.

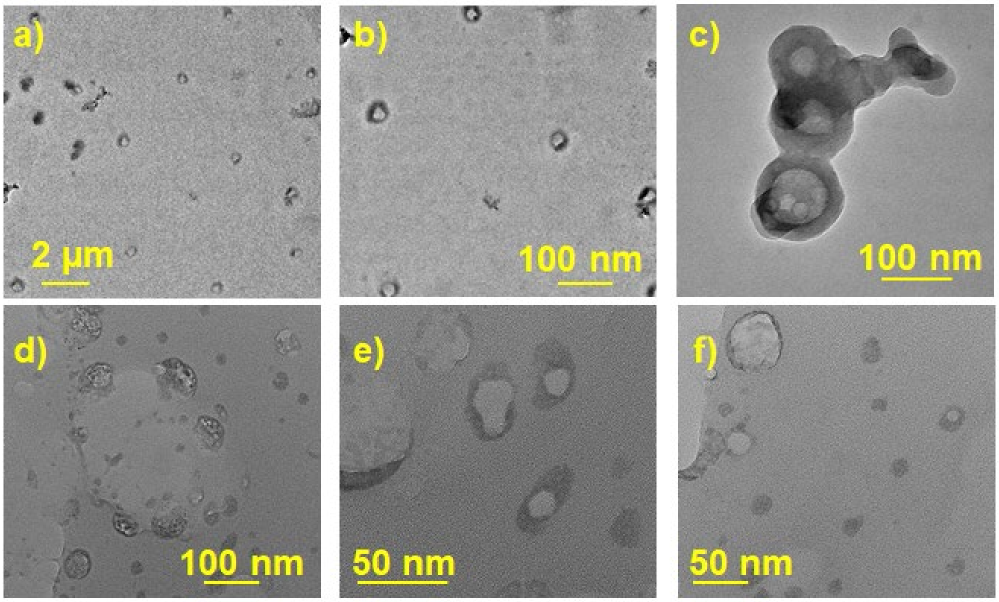

Supplementary Fig. 14. a-f) HRTEM images of TTA-MCDFP COF vesicular particles at various magnifications. Samples were prepared on a holey carbon film on a copper grid. A drop of dilute aqueous particle solution irradiated at room temperature for one hour (365 nm) was applied onto the grid and dried overnight at room temperature.

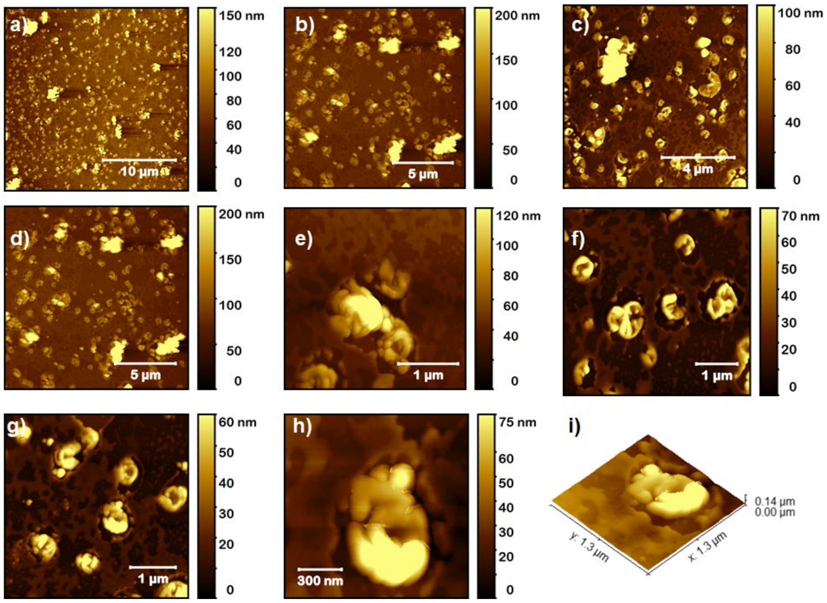

Supplementary Fig. 15. a-i) Tapping mode AFM images of TTA-MCDFP COF vesicular particles at different magnifications. AFM images were acquired by drop casting a dilute aqueous solution of TTA-MCDFP COF onto a Si wafer. The solution was obtained from the dispersion TTA-SPDFP COF after being irradiated with UV light (365 nm) for one hour.

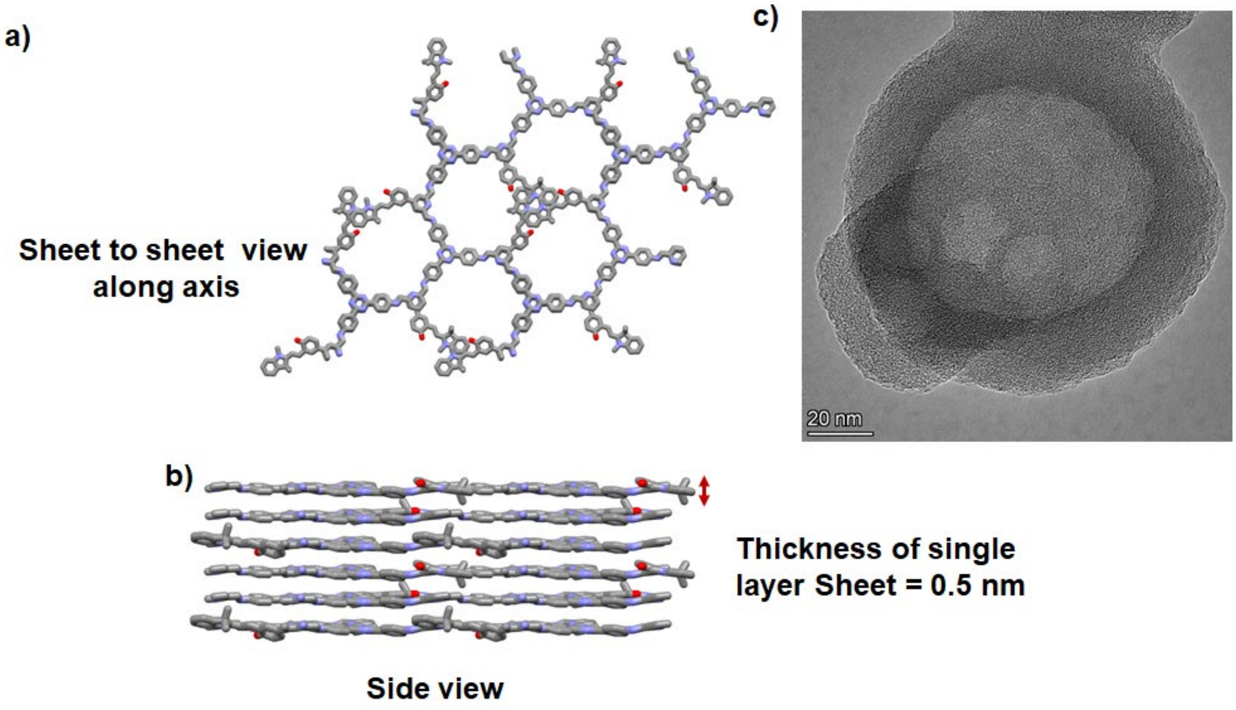

Supplementary Fig. 16. a-b) Computed structure (top and side views) of TTA-MCDFP COF. The single layer sheet thickness is 0.5 nm. c) Zoomed HRTEM image of a hollow vesicle with a shell thickness of ~16 nm.

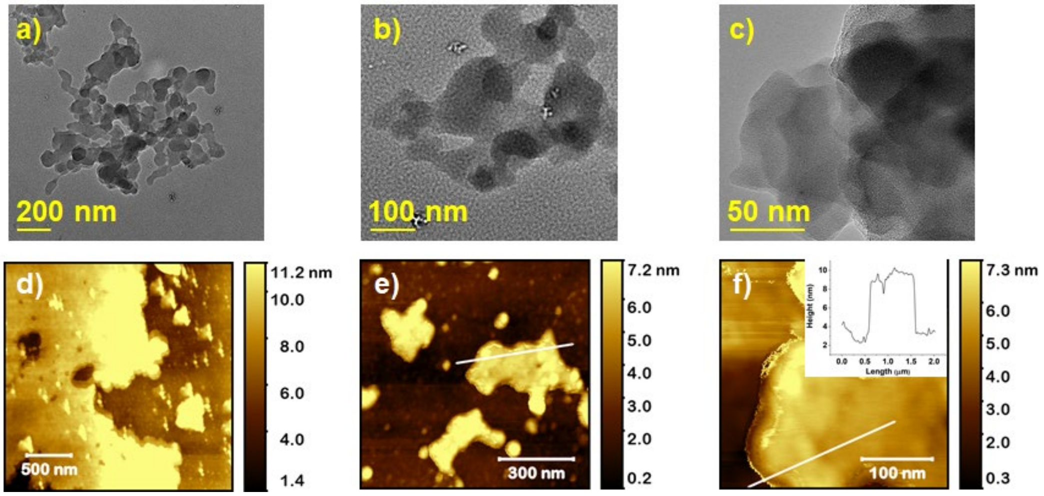

Supplementary Fig. 17. a-c) HRTEM and d-f) AFM images of intermediate-state 2D nanosheets. For HRTEM analysis, the sample was prepared by drop casting the diluted solution onto a holey carbon film on a copper grid. The diluted solution was collected from TTA-SPDFP COF in water at room temperature after 10 minutes of photoirradiation (365 nm), and the AFM grid was prepared from the same solution. AFM images were acquired on a silicon wafer by drop-casting the sample TTA-SPDFP COF. All experiments were performed at room temperature.

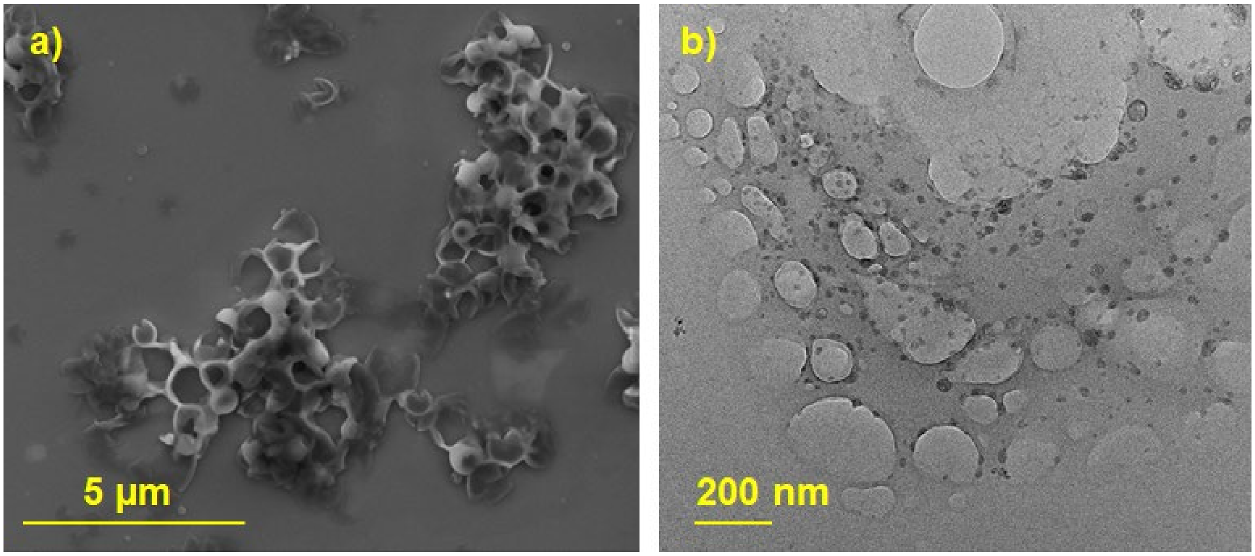

Supplementary Fig. 18. a) SEM and b) HRTEM images of the TTA-MCDFP hollow vesicles. For SEM analysis, the sample was prepared by drop casting the dilute COF solution onto a Si wafer and for HRTEM analysis by drop casting the dilute solution onto a holey carbon film on a copper grid. The solution was collected from the aqueous COF dispersion medium after prolonged UV light irradiation (t = 24 hours). The hollow vesicles showed no further changes in their shape or size after prolonged time. SEM and HRTEM images were taken at room temperature.

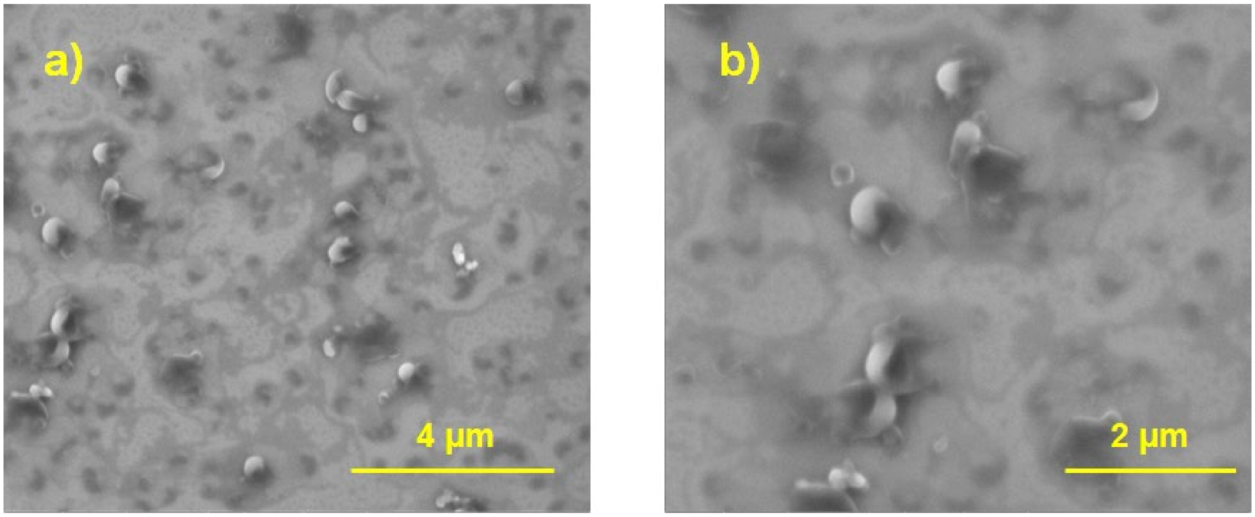

Supplementary Fig. 19. a, b) SEM images of hollow vesicles at different magnifications. The sample was prepared by drop casting a dilute solution of TTA-MCDFP COF, which was stored for one month, onto a Si wafer. The size and shape of the hollow vesicles were not different from those recorded at t = 0. Microscopic analysis was performed at room temperature.

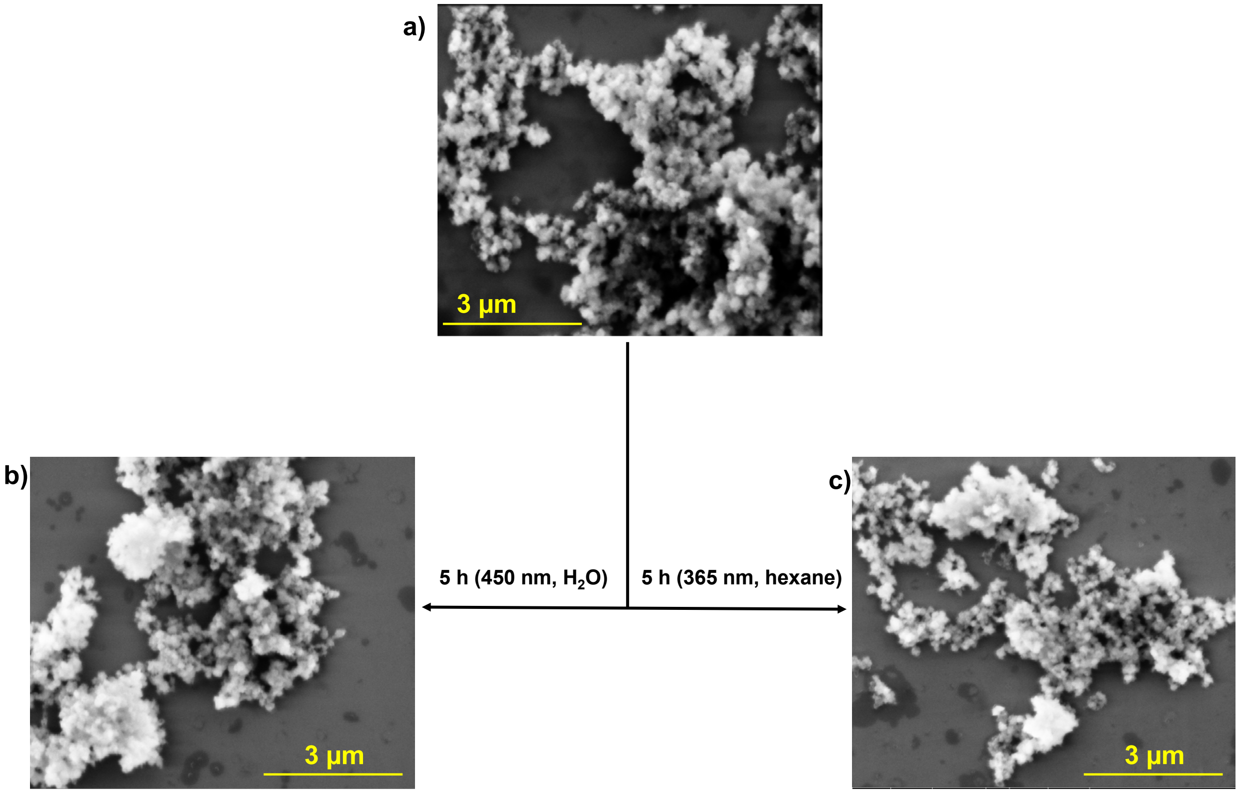

Supplementary Fig. 20. a) SEM image of the as-synthesized TTA-SPDFP COF, the sample was prepared by drop casting the diluted solution of TTA-SPDFP COF on a Si wafer, the solution was taken from the dispersed aqueous solution of TTA-SPDFP COF material without any light irradiation, b-c) SEM images of TTA-SPDFP COF, prepared by the same drop casting method, the samples were taken from b) light irradiated (450 nm, 5 hours) aqueous and c) light irradiated (365 nm, 5 hours) hexane dispersion. Microscopic analysis was performed at room temperature.

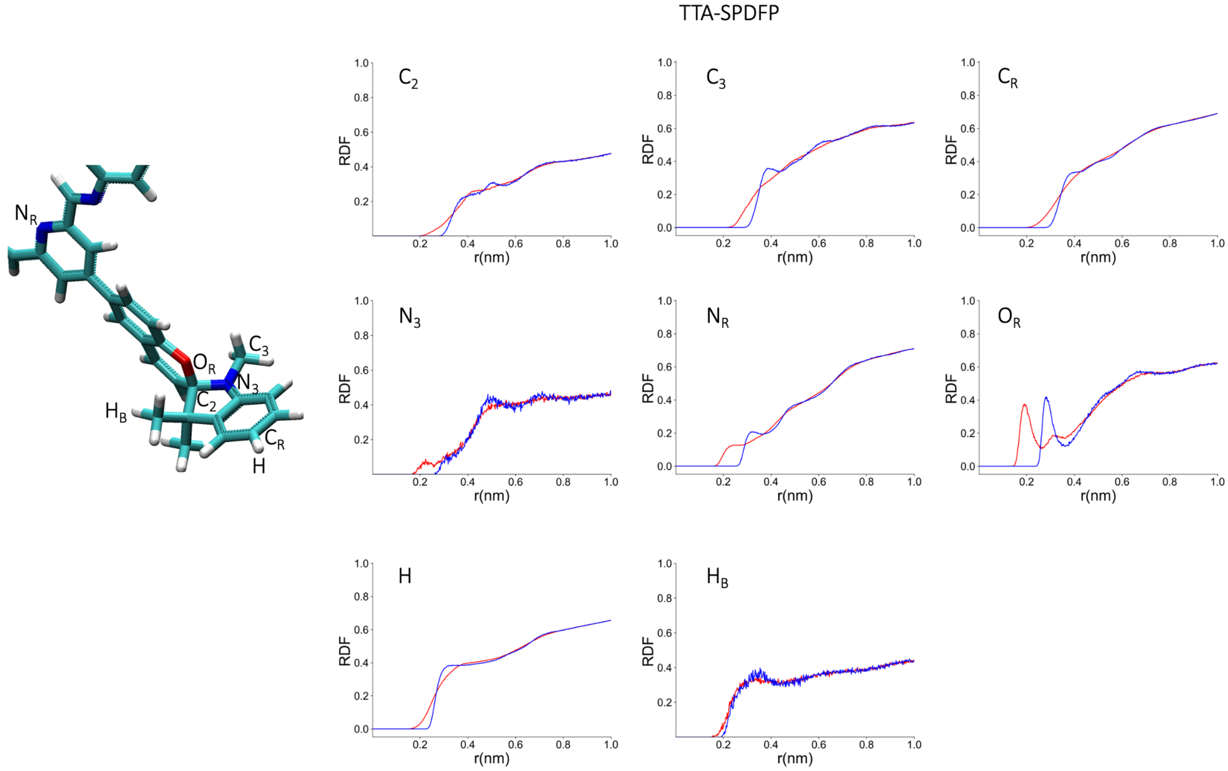

Supplementary Fig. 21. Radial Distribution Function, gαβ (r), of TTA-SPDFP between atom type (α) with water atoms (β). The distance correlations of TTA-SPDFP atoms and water oxygen atom is shown in blue color, and TTA-SPDFP atoms and hydrogen of water is shown in red.

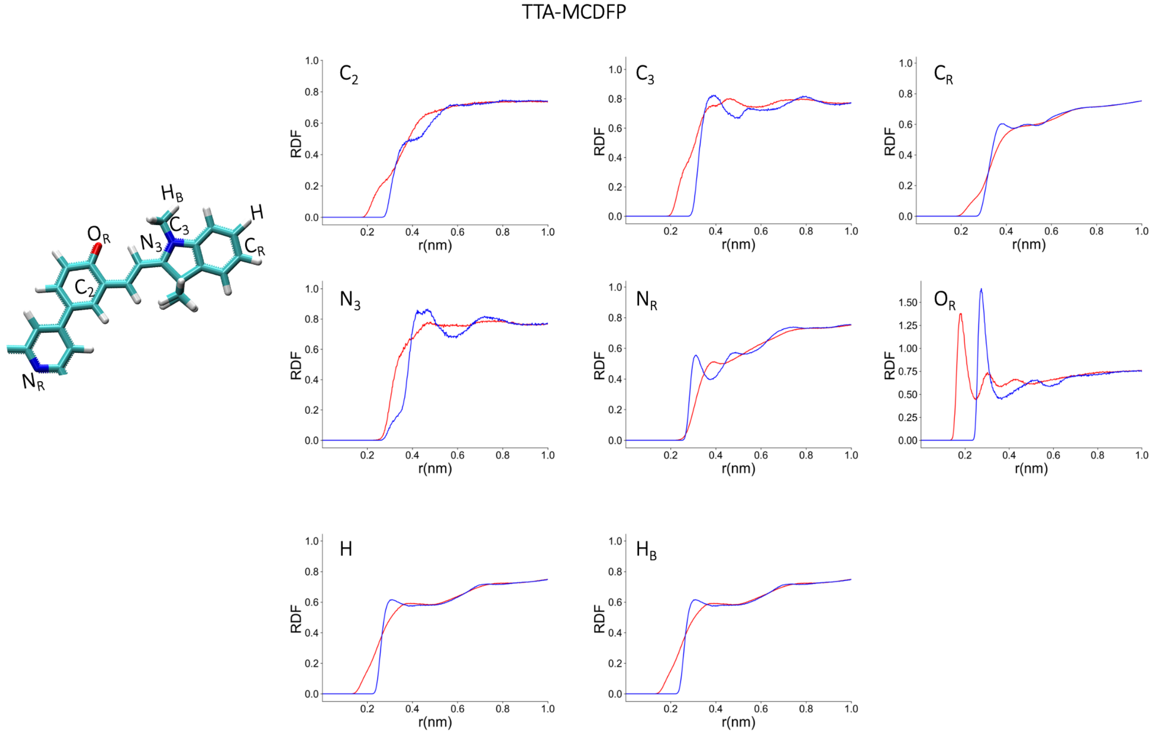

Supplementary Fig. 22. Radial Distribution Function, gαβ (r), of TTA-MCDFP between atom type (α) with water atoms (β). The distance correlations of TTA-MCDFP atoms and water oxygen atom is shown in blue color, and TTA-MCDFP atoms and hydrogen of water is shown in red.

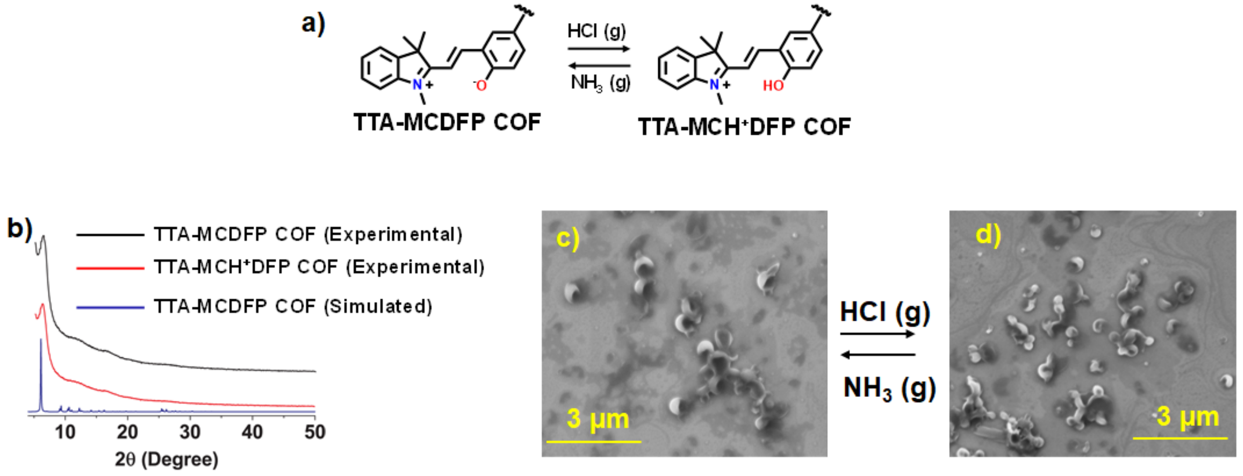

Supplementary Fig. 23. a) Structural changes of TTA-MCDFP/TTA-MCH$^+$DFP COF upon HCl (g) and NH_3 (g) treatment, b) PXRD patterns and c-d) SEM images of TTA-MCDFP/TTA-MCH$^+$DFP COF. The unchanged PXRD and morphology clearly indicated the chemical stability after reversible acid (HCl) and base (NH_3) treatments.

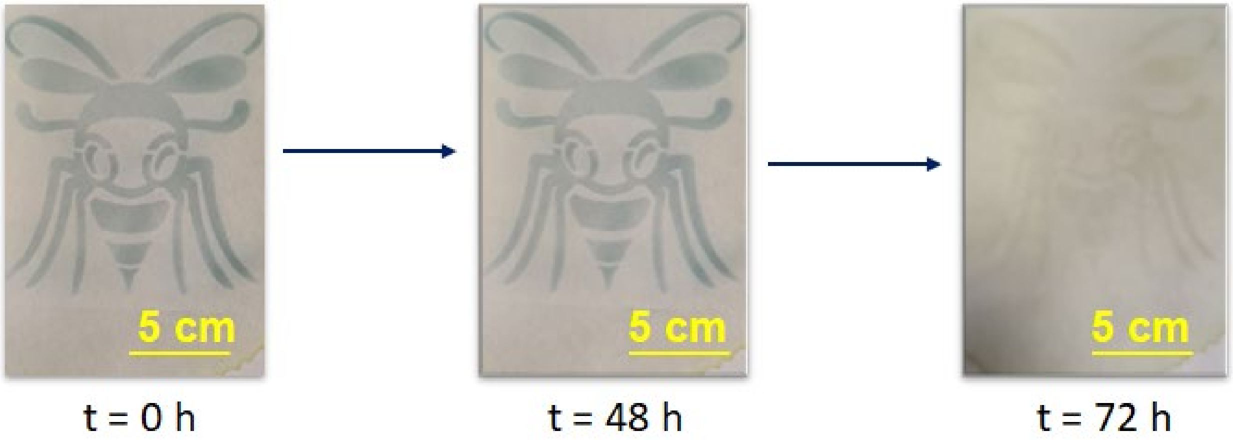

Supplementary Fig. 24. Test of fading stability of printed paper at different time intervals. The long fading time of the printed pattern indicates that TTA-MCDFP can be used for erasable inkless printing.

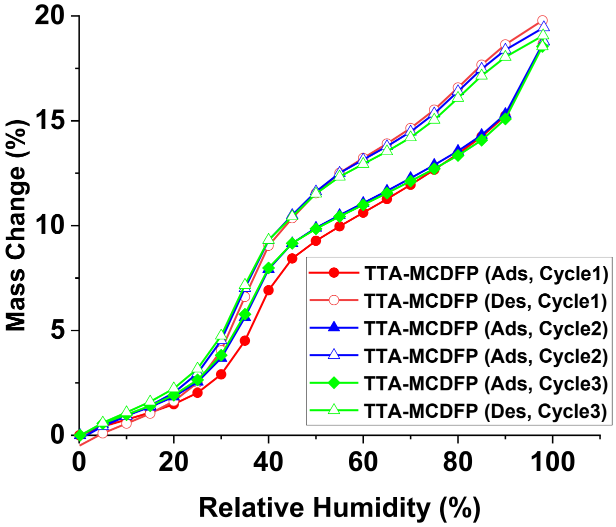

Supplementary Fig. 25. Recyclability and reusability of COFs for water adsorption. Water adsorption–desorption isotherms of TTA-MCDFP COF at three consecutive cycles, measured at 298 K.
