# Supplementary material for: Light-driven self-assembly of spiropyran-functionalized covalent organic framework
Source: Nat Commun. 2023 Jun 23;14:3765. doi: 10.1038/s41467-023-39402-8 (PMC10290075; doi:10.1038/s41467-023-39402-8)
Supplement: Supplementary file 3 — Description of Additional Supplementary Files [file 41467_2023_39402_MOESM3_ESM.pdf]

# Description of Additional Supplementary Files

Supplementary Data 1

**Description:** Simulated structure for TTA-MCDFP COF

**Identifier:** 10.6084/m9.figshare.23451512

Supplementary Data 2

**Description:** Simulated structure for TTA-SPDFP COF

**Identifier:** 10.6084/m9.figshare.23451509
